# Supplementary material for: Patterns of Intron Gain and Loss in Fungi
Source: PLoS Biol. 2004 Nov 30;2(12):e422. doi: 10.1371/journal.pbio.0020422 (PMC532390; doi:10.1371/journal.pbio.0020422)
Supplement: Table S1 — Also available at http://genes.mit.edu/NielsenEtAl/. (4.3 MB ZIP). [file pbio.0020422.st001.zip › NielsenEtAl/html/1208.html]

AN1014.1.NCU01751.1.MG06707.1.FG06012.1


```
 CLUSTAL W (1.82) Multiple Sequence Alignments - Introns Inserted


Sequence 1: NCU01751.1	715 aa
Sequence 2: FG06012.1	709 aa
Sequence 3: MG06707.1	737 aa
Sequence 4: AN1014.1	677 aa
Alignment Length: 738 aa
Number Identitical Residues: 437 aa
Alignment Score (without introns) 20229


MG06707.1 	-MAAQSKPLRPGPAAASALTTARAVVAQLTEHYLAHRTQVSRAVYLTLIVALIHRVRAAV
NCU01751.1	MAPSLSKPVLPV----FRDRTVKGIITQLTSMYLDNRTRISRAVYITLFVALVNRVRHAI
FG06012.1 	-MGSQSKLALP------GDRTLKGIISQLTGLYVSNRTRISRAVWITLFAALVNRIRLAI
AN1014.1  	-MAAQSK--LP------RERSVRQILSSLASLYLRHRTNISRAVYLALFAALAKRIHNAI
          	   : **   *         : : :::.*:  *: :**.:****:::*:.** :*:: *:

MG06707.1 	AEQKAASQREQAARAEQGGTSKALGAEGIADDDEDDTAVVTSSSGGKGKKKRKKVELNRE
NCU01751.1	AEQKAASVRDAAKRAERQGT-------------------TASTSDDALGGKKKKVELNRD
FG06012.1 	SEQKAASVREAAQRAARRGT-------------------TSSGSEETP---RKKVELNRE
AN1014.1  	SEQKAASQRQVDLRR-KPGT---------------------SSLGDGGDKPRKKVGVNRE
          	:****** *:   *  : **                     *         :*** :**:

MG06707.1 	FFRSLLKLVRICVPGWRSKETRLLISHSFFLVVRTLISLKVAAMDGAIVKSLVKGNGKEF
NCU01751.1	FFRSLLRLLKIVVPGWRSKETRLLISHSFFLVLRTLISLKVAAMDGAIVKALVKGNGREF
FG06012.1 	FFRSLLKLLKIVVPGWRSKESRLLMSHSFFLVLRTLISVRVAEMDGAIVKALVKGNGKEF
AN1014.1  	FFRNLARLLKIVIPGWRSKELRLLISHSVFLVLRTLLSLYVAELDGRLVSNLVRGKGKDF
          	***.* :*::* :******* ***:***.***:***:*: ** :** :*. **:*:*::*

MG06707.1 	LKRIVWWMLIAVPATFTNSM0LSYHQAELSLKYRTRLTQHIHDKYLSKLTFYGISALDDR
NCU01751.1	ITRIVYWMLIAVPATFTNSM0LSYHQAELSLKYRTRLTQYIHDKYLSQLTFYGISALDDR
FG06012.1 	LKRIVWWMLIAVPATFTNSM0LSYHQAELSLKYRSRLTQHIHDKYLSNLTFYGISALDDR
AN1014.1  	LLGLVWWM-----------I0LSYHQCKLALSYRKRLTDHIHDKYLSNMTFYAISAWTT-
          	:  :*:**           : *****.:*:*.**.***::*******::***.***    

MG06707.1 	IKNPDQLIAVDVAKFSNS~LAEVYSNLAKPLLDM0TIYTHSLSKSVGGEGVVFMSLLVQL
NCU01751.1	IKNPDQLIAVDVAKFSNS~LAELYSNLAKPILDM0TIYTFSLSKSVGGEGVVFMSLLVQL
FG06012.1 	IKNPDQLIAVDVSKFSNS~LAELYSNLAKPLLDM0TIYTWSLSKSVGGEGVVFMSLLVQL
AN1014.1  	-----------------D2LAELYSNLAKPILDM~MIYNYSLSKSVGGEGLFIMSLLVQL
          	                 . ***:*******:***  **. **********:.:*******

MG06707.1 	SANVMRALTPPFGKYVADEARLEGEFRFQHSRLIDHAEEVALYHGHEAEKNTLDKGYFTL
NCU01751.1	SANVMRALTPPFGKYVAEEARLEGEFRFQHSRLIDHSEEVALYAGHEAEKDTLDKGYFTL
FG06012.1 	SANVMRALTPPFGKYVADEARLEGEFRFQHSRLIDYSEEIALYGGHNAEKDTLDKGYFTL
AN1014.1  	SANVMRALTPPFGKYVADEARLEGEFRFLHSRLIDYCEEIALYHGHEAEKDTLDKGYFTL
          	*****************:********** ******:.**:*** **:***:*********

MG06707.1 	IKHVNYILRRRFYHGFMEDFVIKYFWGALGLLLCSVPVFVKLPGHISMNMGDRTES~FVT
NCU01751.1	IKHVNYILRRRFYHSFMEDFVIKYFWGALGLMLCSVPVFVKLPGQLQMNMGDRTET~FVT
FG06012.1 	IKHVNYILRRRFYHGFMEDFVIKYFWGALGLMLCSVPVFIKMPGHIAMNMGDRTEA~FVT
AN1014.1  	IKHVNRILRRRLYHGFMEDFVIKYFWGALGLILCSVPVFFRISDQVTQTMGDRTES1FVT
          	***** *****:**.****************:*******.::..::  .******: ***

MG06707.1 	NRRMLLSASDAFGRIMFSYREIMELAGYTSRVASLLDVMDDIQAGHFEKKLVSATGIEDN
NCU01751.1	NRRMLLSASDAFGRIMFSYREIMELAGYTSRVSSLLEVMDDIQNGHFEKKLVSSSGTEDN
FG06012.1 	NRRMLLSASDAFGRIMFSYREVMELAGYTSRVATLLDVMTDVQAGHFEKKLVSSSGIEGN
AN1014.1  	NRRMLLSSSDAFGRLMFSYKEISELAGFTARVSSLLDVMDDLLAGRFEKKLVSSASTEEN
          	*******:******:****:*: ****:*:**::**:** *:  *:*******::. * *

MG06707.1 	AAVLKGRGKVVESEDIEFIDV2PIISPNGDVLVKALSFSLKQGDHLLVVGPNGCGKSSLF
NCU01751.1	EAVLKGRGKVVESRDIEFIDV2PIISPNGDVLVKALSFKLRAGDHLLVVGPNGCGKSSLF
FG06012.1 	EAVLKGRGTVHESNDITFIDV2PIISPNGDVLIKALSFTLKHGDHLLVVGPNGCGKSSLF
AN1014.1  	AAVLSGRGIVEESDAIEFTDV~PIVSPNGDVLVRKLTFTVHPGDHLLIVGPNGCGKSSLF
          	 ***.*** * **  * * ** **:*******:: *:*.:: *****:************

MG06707.1 	RILGGLWPVYGGTVYKPPFTSIFYIPQRPYLSRGSLRQQITYPDGLRTVRAKGVSDADLL
NCU01751.1	RILGGLWPVYGGTVHKPPFTDIFYIPQRPYLSRGSLRQQIIYPDGLRTMRAKGVTDADLL
FG06012.1 	RILGGLWPVYGGTVYKPPFHAIFYIPQRPYLSRGSLRQQIIYPDSLRQMRARGITDADLV
AN1014.1  	RILGGLWPVYGGRVKKPRFEDIFYIPQRPYLSRGTLRQQVIYPDGVREMRAKGITDDDLY
          	************ * ** *  *************:****: ***.:* :**:*::* ** 

MG06707.1 	EVLKILNLEHLVDLYPEGWDAEAEWRDVLSGGLQQRVAMARLFYHRPRYAILDECTSSVT
NCU01751.1	AILKVLNLEHLLQLYPEGWDAEAEWRDVLSGGLQQRVAMARLFYHKPRYAILDECTSSVT
FG06012.1 	EYLKILGLEHLPGLYDEGWDAEAEWRDVLSGGLQQRIAMARLFYHRPKYAILDECTSSVT
AN1014.1  	DVLSVVEIASVVDR-PDGWDAEEEWRDVLSIGLQQRIAMARLFYHRPKFAILDECTSSVT
          	  *.:: :  :     :***** ******* *****:********:*::***********

MG06707.1 	LETEKVMYDNAKALGVTLMTVSHRRSLWKYHSRILQFDGQGNYIFTKLDAETRLKLED~E
NCU01751.1	LETEKVMYDNAKALGITLMTVSHRRSLWKYHTHILQFDGQGKFVFTKLDAEKRLELED~E
FG06012.1 	LEIEKAMYDTAKSLGVTLMTVSHRRSLWKYHSHILQFDGQGNYVFTKLDADRRLKLED~E
AN1014.1  	LEIERVMYETAKKLGTTLMTVSHRRSLWKYHKMILQFDGQGGYIFTGLDWEKRMKLED2E
          	** *:.**:.** ** ***************. ******** ::** ** : *::*** *

MG06707.1 	RDDLEAKLRQVPEIERRIAELTAA
NCU01751.1	KEDLDVLLRQVPELERRIAELTAE
FG06012.1 	KEDLEVRLRQVPELERRIAELTAA
AN1014.1  	KEELDLQLRAIPELQKRMAELTST
          	:::*:  ** :**:::*:****:
```
